# Registry or randomization – must it be evidence or could it be coincidence?

**DOI:** 10.1515/iss-2022-0028

**Published:** 2024-06-26

**Authors:** Olga Radulova, Florian Oehme, Sandra Korn, Christian Praetorius, Marius Distler, Jürgen Weitz

**Affiliations:** Department of Visceral, Thoracic and Vascular Surgery, University Hospital Carl Gustav Carus, Technische Universität Dresden, Dresden, Germany; National Center for Tumor Diseases (NCT/UCC), Dresden, Germany; German Cancer Research Center (DKFZ), Heidelberg, Germany; Faculty of Medicine and University Hospital Carl Gustav Carus, Technische Universität Dresden, Dresden, Germany; Helmholtz-Zentrum Dresden – Rossendorf (HZDR), Dresden, Germany

**Keywords:** randomized controlled trial, register trial, register-based randomized controlled trial, surgical research

## Abstract

Over the years, clinical registries and randomized controlled trials gained acceptance. With increasing experience, it was possible to obtain knowledge of benefits and limitations in both designs. During the last years, the research focus was placed on new study concepts such as register-based randomized controlled trials intending to merge the benefits of evidence obtained by RCTs and clinical registers. In this review, we aim to provide an overview of the evolution and the present stage of clinical trials. While doing so, we outline past experience and look ahead toward improving models for high-quality clinical trials.

## The historical evolution from coincidence to evidence

Scientific evidence is always generated based on the underlying problem, anchored in everyday medical practice. To obtain data for treatment improvement, high-quality clinical research is needed. Historically, clinical examinations were carried out already in the 18th century. First data obtained in a clinical study were published already in the 18th century by the physician James Lind after his observations on navy staff and the association of the occurrence of scurvy with the consumption of citrus fruits [1]. In the 1940s, the principle that led to comparability of the studied groups by coincidence was introduced under the term randomization. In this case, the study participants are allocated by coincidence (are randomized) to a treatment group formulated in the study protocol [2]. By comparing the data from both groups, a statement can be made on the effect of the studied treatment. The first randomized controlled trial was conducted in England in 1948 and investigated the therapy of tuberculosis [3]*.* The method of randomly assigning participants to comparison groups became an internationally accepted standard for studies in the 1960s. Those were the first steps toward obtaining scientific certainty, nowadays recognized as evidence, in clinical practice.

In the 1990s, the term evidence-based medicine was first introduced by the Canadian researcher Gordon Guyatt [4]. The aim was to replace the previous practice of treating patients, based on the experience of individual physicians, with a scientifically based approach. Currently, the term evidence-based medicine describes a treatment based on scientific findings, enabling the best possible patient care. Moreover, while representing clinical experience, evidence-based medicine provides the basis for further academic research.

### At the beginning is the question

The rationale for conducting clinical trials is addressing everyday clinical questions related to patient care. One of the central questions is how to reduce complications and mortality while achieving better long-term survival and quality of life. The answer of these questions is very straightforward and clear – by improving therapies, resulting in improved outcome. The critical consideration to identify a medical-scientific problem and subsequently to find a solution, no matter which method is used, signifies the entry into the scientific work for the surgeon.

In this context – if you have a plan, you are closer to success. Whether the results of a study are representative and pave the way for evidence-based therapies depends on optimal planning the study idea. Especially since the following step, deciding which methodology should be used in answering the question raised, inevitably exposes one with the dilemma of any study initiation:

“Is a randomized-controlled trial necessary, or can I achieve a comparable result with an observational study?”

### Must it be evidence?

#### Randomized-controlled trial (RCT)

The randomized, controlled-comparative trial (RCT) still represents the gold standard of scientific evidence for evaluating safety and outcome in patient treatment [5]. The study design allows to compare two treatment groups by randomizing. This is suitable for surgical research (e.g., a new surgical technique with established procedure) as well as for phase III studies for drugs or medical devices.

RCTs allow to test the efficacy and safety of the therapy in a larger and heterogeneous study population in the clinical practice. Like in any human clinical trial, following ethical principles of the World Medical Association declaration of Helsinki and the principles of Good Clinical Practice (GCP), patient safety should be guaranteed [6]; the trials are conducted only after approval by an independent ethics committee and/or official authorities. For RCTs, a primary endpoint is formulated as a null hypothesis based on a medical scientific rationale. The patient groups are comparable and the allocation of patients to the groups is performed by random selection.

The fundamental base for any successful trial is a clearly defined study population and well-defined inclusion and exclusion criteria. Furthermore, the cohorts should be structurally similar, i.e., possible confounders must be avoided or reduced. Confounders are factors that may have an influence on the therapy (gender, age, comorbidity, BMI, and so on) and may be reduced by stratification (allocation of the population into subgroups – e.g., same age).

### RCT benefits

In a randomized-controlled trial, randomization (with or without stratification) allows the equal distribution of confounding factors (BIAS) and unknown influencing variables and relativize the influence of the treatment provider on the choice of therapy [7]. The more extensively these confounding factors were accounted for in the study design, the higher is the internal validity of the study results. Internal validity indicates if the study is well conducted and if it focuses on strong research methods to remove possible alternative statements and validate results.

The smartest and safest method to avoid bias is to distribute all confounders and to stratify the groups. As a result, the confounding variables are minimized by allocating patients by chance into comparable groups. The RCT thus allows a direct conclusion regarding the influence of the analyzed intervention on the primary endpoint (internal validity). Providing good balance between the comparable groups, RCT’s benefit is the strong internal validity due to randomization and stratification. Noteworthy, as stratified randomization is applied to avoid mismatch across groups of factors influencing treatment, it may improve the power in small studies (<400 casenumbers), particularly in cases of high factor effect. This makes stratification advantageous only in small-sized RCTs [8].

**Figure 1:**
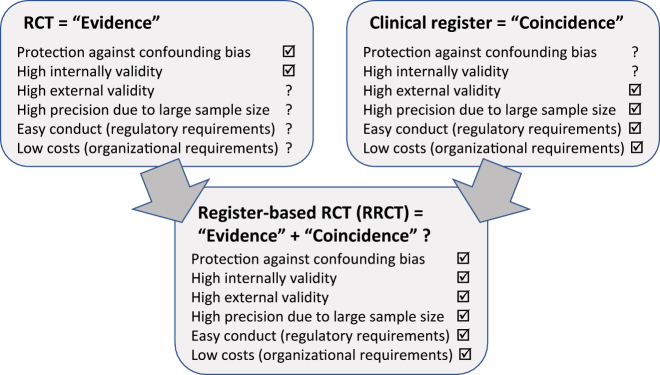
In a modern scientific approach, benefits of clinical registers and RCTs may be merged to reduce complexity of the study process and to enable conducting more high-quality studies with a large number of patients. The register-based RCT combines the advantages of both designs, so the randomized comparison has perfect internal validity. The figure shows the benefits and limitation of the different types of studies.

The internal validity reflects the relationship between cause and effect and thus the causality between treatment and outcome (Figure 1).

Furthermore, publications of RCT results should be in accordance with the CONSORT Statement, providing standardized requirements for reporting [9]. The aim is to provide clear and transparent results to the readers in a standardized methodology.

### RCT Limitations

Nevertheless, RCTs are not flawless and may suffer some disadvantages. Planning and initiation require a profound consideration of endpoints and influencing factors, validity, statistical methodology, funding, and most important – the feasibility of the study. This process is still quite sophisticated and otherwise associated with significant financial burden. Moreover, RCTs are frequently associated with long study duration, and, in doubt, a high rate of trials may end prematurely. The limitations arising throughout this course may lead to disillusionment in young, enthusiastic scientists.

Furthermore, RCTs fall under increasing discussion due to the complexity of their implementation. Many trials have recruitment problems of ever shrinking patient groups by subcategorization (e.g., investigation of a surgical technique in patients stratified by mutation analysis) [7].The high level of selection in the cohorts may lead to less representative results only partially transferable to daily clinical practice.

A significant drawback of RCTs is the external validity, i.e., the translation of the study results to the “real world” population. Thus, the standard and controlled conditions in the study design are only partially transferable to daily practice, not representing the clinical reality [5]. This may limit the number of potentially planned studies [7]. Another restricting factor for the external validity is that the majority of studies are conducted at high-volume centers with a high level of expertise among clinicians and study investigators. The results obtained here are mostly not comparable to those obtained at low-volume institutions and, therefore, not directly transferable to practice [10]. In fact, this also represents a problem for observational studies.

A significant problem for data interpretation is the mismatch between trial protocol and published data. The study by Blumle et al. compares the selection criteria of 52 study protocols with the published data and reports on very disillusioning results. In the published data, 479 of a total of 1,248 eligibility criteria were missing and 163 were modified [11].

## How much coincidence is acceptable?

### Clinical register

A patient register represents an organized system. It uses observational study methodology to collect uniform data and evaluate specified endpoints in defined populations of patients [12].

Depending on the objective of the development, there can be disease-related registries, therapy, or intervention-oriented registries, which are most frequently used for epidemiological data or data on the long-term effects of specific applications.

The aim of the registry is to collect patient-related data in a comprehensive setting to enable epidemiological analyses and quality control. Using nonselected inclusion criteria for patients, the therapy effects are evaluated in clinical practice while subgroups with a better or worse treatment success are identified.

In Germany, several big registries for surgical research were conducted in last year and are available for surgical research. StuDoQ of the German society for general and visceral surgery (DGAV) was established in 2008 as a NOTES (natural orifice transluminal endoscopic surgery) register and has been now expanded to further registries that cover surgery of the rectum, pancreas, stomach, thyroid, and other fields, which makes it currently the biggest registry for surgical research in Germany. Further structures (oncological data registries, registries of associations, routine hospital data, insurance companies, etc.) have been continuously developed in last years.

To improve the quality of clinical registries in Germany, a memorandum for quality criteria on medical registries was drafted in 2010 by a German network in health services research (DNVF) [13]. The Memorandum provides a definition and guidelines for the development of registries and a checklist for assessing the quality of the registers.

### Register benefits

The registries provide results generalizable to a broad range of patients and offer valuable evidence for quality assurance in health care and epidemiology. They make it possible for investigators to obtain records representing the whole underlying population. A crucial point is to collect uniform data standards and definitions, so data truly represent the cases occurring from the underlying population. As a result, the conducted study has a strong external validity. This means that findings may be generalized to some underlying population [14].

Registries are also used as an influential source of knowledge for planning RCTs. In clinical research, such database may help to generate hypotheses for RCTs and to prove the results in long-term outcome.

### Register limitations

Registries are usually unsuitable for the evaluation of comparative treatment effects. Due to the heterogeneous patient population, treatment outcomes cannot be analyzed accurately. This means that, in an observational study, causality between intervention and effect can only be approximated by statistical analyses that limit the influences of confounding variables by specific techniques (e.g., stratified, matched-pair, multiparametric, or propensity score analysis). Furthermore, equality in patient groups is not given in registers, it can be reached only by coincidental allocation (randomization), contributing significantly to avoidance of confounders [15]. A very important issue in the development of clinical registries and their reliability is whether routine data or data selected by specialists are collected.

## Increasing evidence by boosting coincidence?

### Registry-based randomized clinical trials (R-RCTs)

In 1967, two different study designs were reported by Schwartz and Lellouch for “explanatory” and “pragmatic” studies. The first is intended to examine the effect of the therapy, the so-called “efficacy,” using highly selected criteria. The second is designed to study an unselected cohort of patients and so-called “effectiveness” in the clinical routine [16]. To merge both study designs into a relatively new concept in medical research, the concept of registry-based randomized clinical trials was developed. As an attempt to address the challenges of RCTs, an increasing interest has been focused on patient registries as prospective databases providing information on long-term outcomes. The result was the concept of RRCTs, which were designed with the aim of reducing the limitations of the RCTs, by using the benefits of clinical registers. As a consequence, registry-based observational prospective studies as a source of a “real world data” were used to provide data for efficacy and safety. In the first standard manual on this topic, R-RCTs are described as an organized data system, which acquires both population and disease data from observational studies used to evaluate clinical questions [12]. The study design was initially considered to be very promising by increasing the external validity and simultaneously reducing the cost and additional burden. In the last 30 years, a lot of high-quality registries collected vast amount of data, for example, in cardiology (The National Cardiovascular Data Registry (NCDR^®^) of the American College of Cardiology; https://cvquality.acc.org/NCDR-Home), oncology (The Surveillance, Epidemiology, and End Results (SEER) Program of the National Cancer Institute of the United States; https://seer.cancer.gov), NORDCAN – (The Association of the Nordic Cancer Registries), etc.

One of the successful and most often mentioned studies within this new concept is the TASTE (Thrombus Aspiration during ST-Segment Elevation Myocardial Infarction) study from Scandinavia. In this trial, routine thrombus aspiration in ST-elevation myocardial infarction before percutaneous coronary intervention (PCI) vs. PCI alone to reduce mortality in the first 30 days is investigated. Using a very pragmatic approach by implementing a well-established registry (Swedish Coronary Angiography and Angioplasty Registry (SCAAR)) and IT platform (The Swedish Web-system for Enhancement and Development of Evidence-based care in Heart disease Evaluated According to Recommended Therapies-SWEDEHEART), and choosing mortality as a primary endpoint of the trial, the authors were able to randomize 7,244 patients in a short period of 2 years [17]. The study was completed in less than 3 years and was cost-effective with $50 per patient, which is approximately>90 % less compared to an RCT [7], 18].

## Discussion

Currently, we are facing an increasing trend in medical research with so-called “Big Data” – access to as much data as possible, ideally all data (“Real World Data”). Thus, the question arises whether these data collections could not replace the very time-consuming RCTs. Further, heterogeneity may be even encouraging with respect on keeping the data as close as possible to the reality of patient care. Considering current evidence, a trial design with such advantages appears extremely attractive.

Williams et al. showed in their study on the WHO-listed “ClinicalTrials.gov” registry that about 12 % of all studies, a substantial proportion of which are RCTs, had to be terminated prematurely due to problems [19]. The results of an analysis by Chapman et al. are even clearer: In addition to a discontinuation rate of 21 % of all RCTs (81 out of 395 RCTs), only 66 % of the completed studies were published [20]. Almost scandalous was the finding that industry-sponsored studies were published much less frequently, especially when the results did not yield the desired outcome.

Given such conditions, the question of alternatives to systematic RCTs is justified. Certainly, the evidence of RCTs is a convincing argument for this study design; however, large observational studies of the last decade have yielded substantial findings that have had an impact on surgical management [21]–23]. These publications with the greatest scientific impact came from registry studies, with patient recruitment and database, which cannot be achieved by RCTs. Examples of successful registries with several years of activity and substantial scientific contribution are the *SEER* Program, the *German Registry for acute Aortic Dissection Type A (GERAADA)*, or the *NORDCAN.*

GERAADA is a German registry for acute aortic dissection type A, set up by the German Society for Thoracic and Cardiovascular Surgery in 2006. Within 4 years, 2,137 patients were enrolled in the study. Data analysis detected factors influencing the outcome of patients with acute aortic dissection type A, which may be implemented in guidelines to improve treatment results in the clinical practice [24]. Unfortunately, to the best of our knowledge, no register-based RCT so far was conducted in Germany making use of GERAADA or any other large surgical register.

Of course, with the decision for an observational study, there is a substantial risk of bias, which might lead to confounded results. Therefore, registry analyses give only high-certainty results, if the size of the effect is very large and thus cannot be attributed to confounding bias alone. Accordingly, registries may still be more suitable than RCTs for evaluating the safety of interventions, because safety problems with a new intervention may lead to a dramatically increased rate of complications that were seldom or never seen with other interventions. Furthermore, large sample sizes and long follow-up usually allow good detection of safety signals in registries. This, however, requires that registries are comparative in nature (i.e., include more than just one type of intervention). Further methodological research should investigate the quality of registries more precisely to enable future broader application [25], 26].

A critical consideration for the appropriate study design is essential at this stage. However, with increasing bureaucratization in medicine, regimentation in research, and intensification of costs, one thought becomes more and more intuitive:

“Must it be evidence, or could it be coincidence?”

Why not merging both concepts by using the benefit of both and developing a new study design? With increasing experience and considerable recruitment challenges, a shift closer to reality emerged. As a result, R-RCTs are gaining increasing popularity while still aiming to expand the number of clinical trials conducted for the purpose of addressing numerous issues of diagnostic, therapy, and clinical course or long-term outcome.

Still, initial findings are not only considered the desired solution either. Thus, first results were rather disappointing. A meta-epidemiological survey from 2016 analyzed the difference in the estimated treatment effect for mortality between observational studies with routinely collected patient data and randomized controlled studies with a similar question. Authors evaluated 16 observational studies and 36 RCTs and found in 31 % of the 16 clinical questions a deviation in the direction of treatment between both study groups. Finally, the summary relative odds ratio shows that analyses of observational studies overestimate treatment effects on mortality by 31 % on average, showing that in general register or electronic patient data are not suitable for the analysis of a relationship between cause and effect in treatment, and should therefore be used with caution as a basis for clinical and system-relevant decisions [27].

However, due to strict eligibility criteria in this meta-epidemiological survey, the number of registry studies was limited. Furthermore, registries included a wide range of data, thus sources differ in completeness and validity. The authors concluded that routine data analyses can systematically and substantially overestimate treatment effects. Nevertheless, comprehension of the risk of bias is not always trivial. It is possible to reduce confounding by applying propensity score methods [27].

According to Li et al., R-RCT related challenges concern the quality of the registries rather than the study methodology [28]. If the clinical registries in the future are going to be used more often as a basis for RRCTs, completeness and correctness of the data should be taken much more into consideration. External quality control, education, training, and improvement of IT-platforms may improve the accuracy of the registry database. The reliability and validity of the registries is undoubtedly linked to a governmental legal and financial support, which is crucial for the establishment of a good study infrastructure. In Sweden and Denmark, data are simply linked via personal identification numbers, which results in nearly perfect registry completeness [29]. Furthermore, the legislative framework allows the inclusion of administrative data for statistical and research purposes. Since the data from clinical registries focus on long-term outcomes, such as survival and mortality, reliable, ongoing funding is also a key factor for the quality of the database. As a result, R-RCTs are more frequently conducted in these countries and this could offer Germany some orientation. The history of population-based cancer registration in Germany shows that national efforts are needed to establish high-quality registries, like in the United States, United Kingdom, and Scandinavia [30].

## Conclusion

Therefore, to answer the question “Must it be evidence, or could it be coincidence?”, it is necessary to focus on coincidence encountering evidence and support answers to still outstanding clinical questions in daily life. The establishment of high-quality clinical registries based on a standardized approach with the aim to establish complete and informative prospective databases could be the first step in shifting coincidence to evidence as well as to reduce the challenges of recruitment. This may be an exceptional possibility for a new approach in clinical research, especially in situations in which it is impossible to gain new evidence by RCTs, which will continue to generate the highest level of evidence.
